# A coral-algal phase shift in Mesoamerica not driven by changes in herbivorous fish abundance

**DOI:** 10.1371/journal.pone.0174855

**Published:** 2017-04-26

**Authors:** Jesús Ernesto Arias-González, Tak Fung, Robert M. Seymour, Joaquín Rodrigo Garza-Pérez, Gilberto Acosta-González, Yves-Marie Bozec, Craig R. Johnson

**Affiliations:** 1 Laboratorio de Ecología de Ecosistemas de Arrecifes Coralinos, Departamento de Recursos del Mar, Centro de Investigación y Estudios Avanzados I.P.N.-Unidad Mérida. Carr. Ant. Progreso Km. 6, A.P. 73 Cordemex, Mérida, Yucatan, Mexico; 2 National University of Singapore, Department of Biological Sciences, 14 Science Drive 4, Singapore, Singapore; 3 Centre for Mathematics & Physics in the Life Sciences & Experimental Biology, & Department of Mathematics, University College London, London United Kingdom; 4 Unidad Multidisciplinaria de Docencia e Investigación Sisal, Facultad de Ciencias, Universidad Nacional Autónoma de México, Puerto de Abrigo S/N, Sisal Yucatán Mexico; 5 Unidad de Ciencias del Agua. Centro de Investigación Científica de Yucatán A.C. Calle 8 no. 29 Mza 39 SM 64. Cancún. Q. Roo. C.P. México; 6 Marine Spatial Ecology Lab, School of Biological Sciences & Australian Research Council Centre of Excellence for Coral Reef Studies, University of Queensland, St. Lucia, Queensland, Australia; 7 Institute for Marine & Antarctic Studies, Private Bag 129, University of Tasmania, Hobart, TAS, Australia; University of California Santa Cruz, UNITED STATES

## Abstract

Coral-algal phase shifts in which coral cover declines to low levels and is replaced by algae have often been documented on coral reefs worldwide. This has motivated coral reef management responses that include restriction and regulation of fishing, e.g. herbivorous fish species. However, there is evidence that eutrophication and sedimentation can be at least as important as a reduction in herbivory in causing phase shifts. These threats arise from coastal development leading to increased nutrient and sediment loads, which stimulate algal growth and negatively impact corals respectively. Here, we first present results of a dynamic process-based model demonstrating that in addition to overharvesting of herbivorous fish, bottom-up processes have the potential to precipitate coral-algal phase shifts on Mesoamerican reefs. We then provide an empirical example that exemplifies this on coral reefs off Mahahual in Mexico, where a shift from coral to algal dominance occurred over 14 years, during which there was little change in herbivore biomass but considerable development of tourist infrastructure. Our results indicate that coastal development can compromise the resilience of coral reefs and that watershed and coastal zone management together with the maintenance of functional levels of fish herbivory are critical for the persistence of coral reefs in Mesoamerica.

## Introduction

Coral-algal phase shifts in which coral cover declines to low levels and is replaced by algae [1,2,3] challenge the management of coral reefs worldwide [4,5], including in Mesoamerica [6]. Phase shifts may be caused by many factors, encompassing both episodic pressures of short duration and chronic pressures of long duration [2]. Following the Reefs At Risk assessment [7], among the most important local and global threats identified are overfishing, pollution, coastal development and climate change. Climate change combined with local stressors was identified as threatening 75% of the world’s coral reefs, and overfishing was identified as the most prevalent local threat, affecting around 55% of the world’s reefs [7]. In the scientific literature on coral-algal phase shifts, overfishing is commonly cited as a key underlying driver (e.g., [1,3,8]). This has motivated coral reef management responses that include restriction or prohibition of fishing effort in designated areas [9] and efforts to limit consumer demand for ecologically important species (e.g., [10,11]).

However, as a number of studies have highlighted [12–17], the threat to coral reefs from watershed pollutants is potentially as important as overfishing. Indeed, dynamic models of specific reef sites in the Philippines indicated that the combined effects of nutrification and sedimentation were more important drivers than overfishing in causing coral decline and increased algal cover, while improving water quality rather than managing fishing was the most expedient way to recover coral cover [17]. In general, watershed pollution has been difficult to manage because of the wide variety of pollutants that collectively span a large area and because of the high cost of treating polluted water [12]. The importance of this threat reflects rapid human development on many tropical coasts, which continues to have important impacts on coral [18]. In Mesoamerica the tourism industry has expanded since the early 1970s to encompass large swathes of tropical coastal zones, including most of the Mexican Caribbean [19]. This has been associated with marked impacts on coastal systems that include coral reefs [20,21]. Coastal development and associated watershed pollution [22–27] may have been key drivers behind a notable decline in coral cover and complementary rise in macroalgal cover in the region [21, 28–30].

Previous empirical and theoretical studies on reefs in the Caribbean have emphasized how a reduction of grazing pressure, caused by a collapse of the herbivorous urchin *Diadema antillarum* and overfishing of herbivorous fishes, can increase the susceptibility to coral-algal phase shifts [1,31,32]. Critically, evidence suggests that transitions from coral to algal-dominated reefs are likely to involve a pronounced hysteresis [32,33]. In this case, it is difficult to reverse the transition and achieve recovery of coral–returning an algal-dominated reef to environmental conditions (including herbivore levels) that previously supported abundant coral cover may be insufficient to enable coral recovery [32,33]. Phase shift dynamics have important implications for managing reefs, and full knowledge of the range of factors that determine coral reef resilience and the circumstances in which hysteresis arises is clearly critical for informed management. Here, we first present results of a dynamic process-based model demonstrating that increased nutrients and sediments, which are common effects of coastal development and watershed-based pollution, can be important drivers of coral-algal phase shifts on Mesoamerican reefs in addition to fishing of herbivores. We then complement the modeling analyses with a case study from coral reefs off Mahahual in Mexico, showing that these reefs have undergone a shift from coral to algal dominance with little change in herbivore biomass, but which is coincident with an increase in tourism and associated coastal development. This suggests that the main driver of the shift is increased coastal development arising from the growth of tourism and urban development in the area, reflecting a problem that appears to be undermining water quality and harming the coral reefs in the Mesoamerican region as a whole [22–27,30,34].

## Methods

This study does not require an ethics statement as we did not manipulate any animal or plant.

### Study site

**Fig 1:**
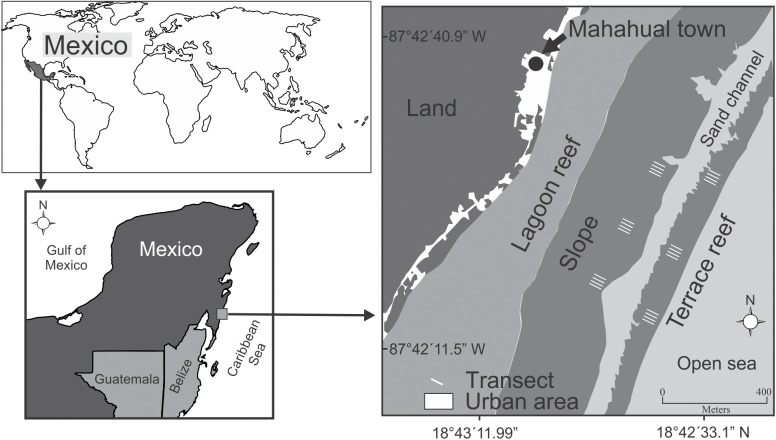
Location of study sites on reefs at Mahahual. 12 transects were deployed at both the slope and terrace (indicated by thin white lines on the figure).

The coral reef system of Mahahual is located in the northern part of the Mesoamerican Barrier Reef System in the state of Quintana Roo, within the touristic area of the Mexican Caribbean known as Costa Maya (Fig 1). This reef system is very close to the coast; the reef crest is ca. 150 m from shore and the reef slope and terrace extend to 0.5–1.0 km from shore. In 2000, the condition of Mahahual’s reef system was reported as relatively good in the Mexican Caribbean [25], but in 2000–2001 a pier was constructed to receive large tourist cruise ships in the northern part of the reef. This stimulated construction of related urban and tourism infrastructure, including restaurants, artificial beaches, navigation channels and hotels. These expansions together with hurricanes and bleaching events are coincident with a coral-algal phase shift in this system [18].

### Dynamic model and analysis

We used the coral-reef benthic model of Fung et al. (2011) [33], which describes the dynamics of three major functional groups–scleractinian (hard) corals, dense turf algae and macroalgae–competing for space on a reef substratum. We first provide a brief description of the model and its parameterization, and then we explain how we use the model to examine the typical effects of top-down and bottom-up stressors on coral and algal covers for Mesoamerican reefs.

**Fig 2:**
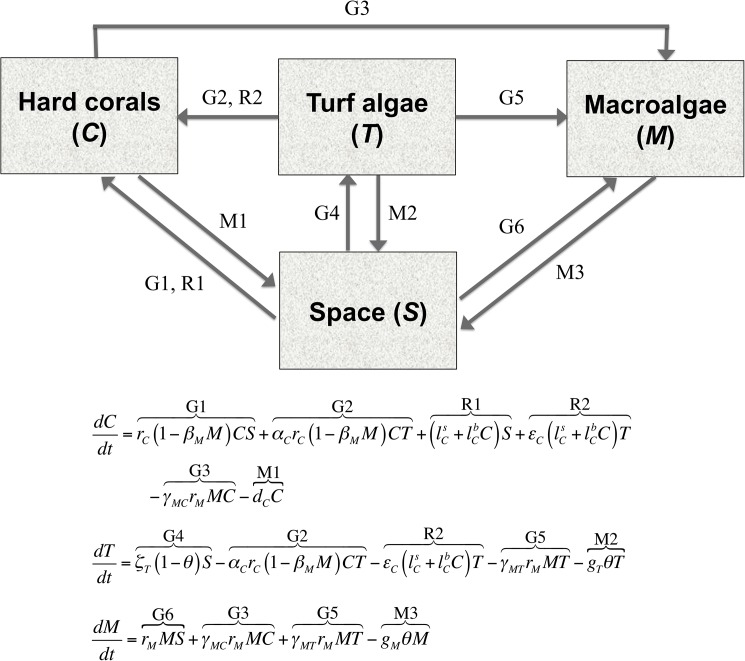
Schematic diagram of the dynamic coral-reef benthic model used. The boxes show the dynamic variables in the model, which are the proportional covers of the three functional groups modeled–hard corals, (dense) turf algae and macroalgae–and space (*C*, *T*, *M* and *S*, respectively). Arrows from one box to another represent conversion of one group to another via the dynamic processes modeled. The processes are grouped into three categories, pertaining to growth (G), recruitment (R) and mortality (M). Underneath the boxes and arrows diagram is the set of differential equations specifying how the variables change over time. There is no equation for *S* because it is a dependent variable that is determined by 1–*C*–*T*–*M*. Terms in the equations represent the dynamic processes modeled, and are numbered to match the corresponding arrows in the diagram. *r*_*C*_ and *α*_*C*_*r*_*C*_ are the rates of growth of hard corals over space and turf algae, respectively, in the absence of macroalgae; $l_{C}^{s}$ and $\varepsilon_{C}l_{C}^{s}$ are the exogenous rates of recruitment of hard corals over space and turf algae, respectively, whereas $l_{C}^{b}$ and $\varepsilon_{C}l_{C}^{b}$ are the corresponding endogenous rates of recruitment; *d*_*C*_ is the mortality rate of hard corals from processes other than overgrowth; ***ζ***_*T*_ is the maximum growth rate of turf algae in space, which is decreased by grazing, measured by the grazing effort *θ* that takes values from 0 to 1; *g*_*T*_ is the maximum grazing rate on turf algae; *r*_*M*_, *γ*_*MC*_*r*_*M*_ and *γ*_*MT*_*r*_*M*_ are the growth rates of macroalgae over space, hard corals and turf algae, respectively; *g*_*M*_ is the maximum grazing rate on macroalgae; and *β*_*M*_ is the negative effect of macroalgae on the growth rate of hard corals. Further details of these parameters and the equations are found in Fung and others (2011) [33].

Dynamics of the proportional covers of the three model groups are represented by a set of ordinary differential equations that represent key ecological processes contributing to their growth, recruitment and mortality. The proportional cover of hard corals increases due to lateral growth over space [35], although the rate of growth is reduced by metabolic costs imposed by contact with macroalgae [36]. Cover of hard corals also increases from establishment of recruits produced by hard corals in the modeled reef area (endogenous recruitment) and hard corals on other non-modeled reef areas (exogenous recruitment) [37, 38]. Coral recruits can establish on ‘free space’ (e.g. substratum supporting microturf) and on dense turf algae at a lower rate [39]. Mortality of hard corals occurs due to stressors such as sedimentation and disease [40], resulting in decreases in proportional cover. Dense turf algae consist mainly of filamentous algae [41] and can arise from growth of algal propagules settling onto ‘space’ [42], thus increasing their proportional cover. On the other hand, dense turf algae can be overgrown by hard corals [43] and macroalgae [32], resulting in a decrease in their proportional cover. Herbivorous fish and urchins exert grazing pressure on the palatable turf algae [44], again decreasing their proportional cover. Macroalgae are distinguished from turf algae by their greater thallus size and structural complexity [41], allowing them to laterally overgrow space, hard corals, and turf algae [35,36,45,46]. Recruitment of macroalgal propagules is assumed to be largely localized [47], and is thus conceptualized as part of the lateral growth process. As is the case for dense turf algae, the proportional cover of macroalgae decreases due to grazing [48]. Fig 2 is a schematic diagram of the model, summarizing the functional groups and how they are dynamically linked by the key ecological processes modeled. Further mathematical and biological details of the model are provided in Fung et al. (2011) [33].

We derived model parameter ranges pertaining to a pristine reef using the methodology of Fung et al. (2011) [33]. This methodology involves estimating the parameters of the modeled functional groups using empirical data from surveys and experiments [33]. Because of our focus on Mesoamerican reefs in the present study, we used the same dataset as that in Fung et al. (2011) [33] except that data from outside the western Atlantic were discarded where possible. Specifically, if the range of a parameter could be derived using just data from the western Atlantic in the dataset, then we did so and disregarded any data from the Indo-Pacific. However, if data from the western Atlantic were insufficient to derive the range of a parameter, then necessary data from the Indo-Pacific were used. Following this procedure, only four out of the 14 parameter ranges required data from the Indo-Pacific for their derivation. Thus, the parameterized model reefs are interpreted as broadly representative of pristine Mesoamerican reefs. S1 Table lists all the model parameters and the locations pertaining to the data used for their parameterization, together with the corresponding references. Full details of the parameterization methodology can be found in Appendix B of Fung et al. (2011) [33].

To quantify the effects of key top-down and bottom-up processes in driving coral-algal phase shifts, (1) 10,000 pristine model reefs were constructed by randomly sampling the parameter ranges according to independent uniform distributions, and then (2) fishing, nutrification and sedimentation were applied in isolation and in all combinations to each model reef. The effects of the three stressors on model parameters were taken from Fung et al. (2011) [33], who derived the effects based mainly on empirical measures from surveys and experiments [49–53]. Specifically, fishing was modeled as decreasing grazing pressure on the reef (potentially down to zero, corresponding to removal of all herbivores); nutrification (addition of increased nutrients) was modeled as increasing the growth rates of both turf algae and macroalgae by up to four times; and sedimentation was modeled as increasing the coral mortality rate by up to three times, decreasing coral recruitment rates by up to a factor of 0.6 and decreasing the coral growth rate by up to 50% (for more details, see Fung et al., 2011 [33]). The severity of each stressor was expressed as a proportion of the maximum effects, such that severity ranged from 0 (e.g., for fishing, this would correspond to no change in grazing pressure) to 1 (e.g., for fishing, this would correspond to a decrease in grazing pressure to zero). In each application of a stressor, the severity was randomly determined according to a uniform distribution. After application of a stressor or set of stressors to a model reef, equilibrium covers of each benthic group were recorded. These covers were subsequently averaged across the 10,000 model reefs. The analyses described here are an advance on those in Fung et al. (2011) [33] because of the use of a population of 10,000 model pristine reefs rather than just one, and the application of stressors with random degrees of severity rather than a fixed degree of severity. This represents a more comprehensive exploration of parameter space and hence assessment of how the stressors could affect coral and algal covers.

For each stressor scenario, we also used our model to quantify the potential for multiple stable equilibria and hence discontinuous phase shifts with hysteresis. Here, multiple stable equilibria refer to the simultaneous emergence of a coral-dominated equilibrium state and an algal-dominated equilibrium state under a fixed set of parameter conditions [32]. A region of parameter space exhibiting multiple stable equilibria results in hysteresis, whereby recovery of corals from the degraded algal-dominated state requires stress to be reduced to levels lower than the threshold that triggered the shift from coral- to algal-dominance in the first place [32,33]. We randomly sampled the parameter space corresponding to each scenario and then determined the percentage of parameter sets exhibiting multiple stable equilibria. This is important in helping to resolve the issue of the likely prevalence of discontinuous versus continuous phase shifts, with and without hysteresis respectively. Fung et al. (2011) [33], which used the same model, did not quantify the likelihood of multiple stable equilibria in terms of the proportion of the feasible parameter space.

### Sampling design and data collection

For the empirical component of our study, data describing benthic species on the Mahahual reef system were obtained in November in the years 1997, 1998, 1999, 2000, 2005, 2006, 2007, 2008, 2009 and 2010. In the years 2000, 2005, 2006, 2007, 2008, 2009 and 2010, data on the abundance and size of fishes were also obtained. A balanced design was used with two spatial scales (two geomorphological units (GUs) as ‘reef slope’ and ‘terrace’, and 12 transects within each GU) and a time scale encompassing 1997 to 2010 [29]. Fish and benthic species were censused using 50 x 2 m belt transects at depths supporting greatest development of *Orbicella/Montastraea*-dominated habitat, namely at ~12 m on the reef slope and ~18 m on the terrace. Within each GU, four transects separated by 50 m were taken at each of three fixed sites (Fig 1). At each site, the fish and benthic communities were assessed by SCUBA diving. For each transect, two assessments were performed, the first visually recording reef fishes >15 cm and the second video-recording the benthos. For our analysis, we have used only biomasses from the families Scaridae and Acanthuridae, which comprise most herbivorous fish biomass. More specifically, we followed the methodology as described by Acosta-González et al. (2013) [29]: “first, four transect lines separated by 50 m were positioned across each fixed sampling site, and then, two censuses were performed, the first recording reef fishes and the second video recording the benthic communities. Two experienced observers, one for the years 2000, 2005, 2006 and 2007 and the other for 2008, 2009 and 2010 conducted visual censuses for all years of sampling. Using the same sampling protocol and a standardized record of species richness, abundance and sizes minimized the bias between observers. We recorded only the species that corresponded to pelagic, demersal and benthic fish, as those are the most conspicuous species that determine the ‘‘visible” fish assemblage structure. We did not include cryptic fish species, as they take too long to count accurately within a transect. The difficulty of visually detecting small-bodied fish is well known and may produce underestimates in the abundance of small cryptic fish such as Gobiidae, Apogonidae, and Blenniidae”. We converted fish size (length *L*) to biomass (wet weight *W* in kg/100 m^2^) using the allometric equation *W* = *aL*^*b*^. The constants for each species, *a* and *b*, were obtained from FishBase (www.fishbase.org) or, if the species in question was not logged in FishBase, different sources from the Caribbean [54,55] or from a species with a similar shape. For each species, total biomass per census was estimated as the average weight multiplied by the abundance [56]. The benthic community was surveyed with underwater video camera at a distance of ~40 cm above the substratum along each 50 m transect [29]. The camera was held ~50 cm to the side of the transect and perpendicular to the substratum.

There was no need for us to obtain specific permission for our field activities, because Mahahual reef is not a protected area and we did not perform any intrusive techniques in relation to the studied fauna and flora.

Once in the laboratory, each video transect was sub-sampled on a computer screen by selecting 40 frames at random, each with 13 systematically dispersed points in a regular grid, totaling 520 points per transect. The benthos under each sampled point was identified to the lowest taxonomic group and life form possible. Benthic organisms were then grouped into three major categories: scleractinian corals, fleshy macroalgae and dense turf algae, and other types of benthic cover. The cover of scleractinian corals was used as a measure of total coral cover, whereas the cover of fleshy macroalgae and dense turf algae was used as a measure of total (macro)algal cover.

Cruise ship passenger arrivals per year at Mahahual were used as a proxy for coastal development, and data were obtained from Anuarios estadísticos de los puertos de México, Secretaria de Comunicaciones y Transportes (SCT) and Administración Portuaria Integral de Quintana Roo, S. A. de C. V. Coordinación de Planeación y Estadística.

### Statistical analysis

Differences in the size (length), abundance and biomass of roving herbivorous fish considered (scarids and acanthurids separately and combined) in different years were evaluated with a permutation-based multivariate analysis of variance (PERMANOVA) using the multivariate statistical analysis package PRIMER-e v.6.1.16 + PERMANOVA v.1.0.6 [57]. Size, abundance, and biomass were each transformed a priori using sqrt(*x*+1), where *x* is size, abundance or biomass. A two-way crossed design was used, where each of the transformed variables was crossed with a factor “year” (seven levels: 2000, 2005, 2006, 2007, 2008, 2009, 2010). After the PERMANOVA, an a posteriori analysis was performed using a pairwise comparison of each variable between every unique pair of years. The permutation method chosen was the method of residuals over the reduced model. Monte Carlo permutation tests were conducted in which a total of 10,000 permutations were performed, with the sum of the coefficients of the fixed effects set to zero [57].

## Results and discussion

**Fig 3:**
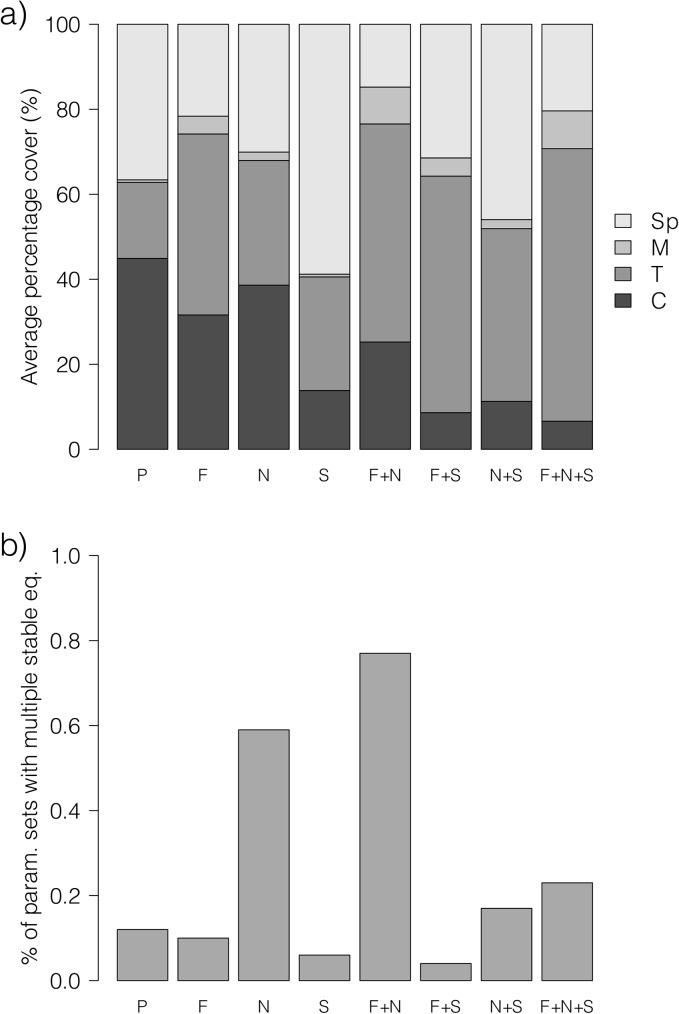
Predicted effects of fishing, nutrification and sedimentation on phase shift potential. a) Average equilibrium percentage covers for pristine Mesoamerican model reefs, subjected to different scenarios: no anthropogenic stress, corresponding to a pristine reef (P); fishing (F); nutrification (N); sedimentation (S), and all combinations of the three types of stressor. Percentage covers are shown for scleractinian corals (C), dense turf algae (T), macroalgae (M) and space (Sp). For this analysis, 10,000 pristine model reefs were generated using the most complex benthic model of Fung and others (2011) [33] by randomly sampling parameter ranges based mainly on empirical measurements on western Atlantic reefs. Fishing, nutrification and sedimentation were then applied as described in the text, with the severity chosen randomly. b) For the same model and scenarios in a), the percentage of parameter space resulting in multiple (two) stable equilibria, indicating the potential for discontinuous phase shifts. For each scenario, the percentage was calculated from 10,000 parameter sets sampled randomly from the corresponding parameter space.

The parameterization of our Mesoamerican benthic model resulted in the same parameter ranges as in Fung and others (2011) [33], except that the upper limit of the exogenous coral recruitment rate (arising from recruits produced by spawning corals on non-modeled reef areas), $l_{C}^{s}$, is now 0.0002 yr^-1^ instead of 0.01 yr^-1^; the upper limit of the endogenous coral recruitment rate, $l_{C}^{b}$, is now 0.05 yr^-1^ instead of 0.5 yr^-1^; and the lower limit of the parameter measuring the negative effects of macroalgae on coral growth, *β*_*M*_, is now 0.4 instead of 0.2. Random sampling of the parameter space resulted in a set of 10,000 pristine model reefs with an average equilibrium coral cover of 45% (Fig 3A). This is near the upper end of the observed range of 20–40% coral cover considered to be representative of healthy reefs in Mesoamerica [58]. When we applied the stressors of fishing, nutrification and sedimentation in isolation and in all combinations to the pristine model reefs, average equilibrium coral covers declined under each scenario, with concomitant increases in average equilibrium algal covers (Fig 3A). The results clearly indicate that a coral-algal phase shift can occur without fishing; under sedimentation alone, average coral cover decreased to 14% and average total algal cover (turf algae plus macroalgal cover) increased from 18% to 27%, and under the combined effects of nutrification and sedimentation, average coral cover decreased further to 11% and average total algal cover increased further to 43%. While average macroalgal cover was low under each scenario (<10% for the pristine scenario and seven scenarios with added stress), macroalgal cover values for individual runs under each scenario always encompassed a wide range of at least 0–81% macroalgal cover, reflecting strong high non-linearity in macroalgal dynamics with changing parameter values. Importantly, this represents the range of macroalgal covers found on real reefs in Mesoamerica [1,28,59]. Under each stressor scenario examined, the percentage of parameter sets yielding multiple stable equilibria was <1%, supporting the view that discontinuous phase shifts are rare relative to continuous ones (Fig 3B; [60]). This bodes well for management intervention because in the case of a continuous phase shift, all else being equal the affected reef would return to high coral cover once issues related to water quality and overfishing are rectified. Nevertheless, our results indicate that discontinuous phase shifts are possible and nutrification in particular was found to increase the probability of multiple stable equilibria and thus hysteresis by a factor of around five, when acting in isolation, or a factor of around six, when acting together with fishing (Fig 3B).

Together, these modeling results emphasize the importance of bottom-up stressors in precipitating phase shifts in addition to the importance of top-down stressors that have been the focus of previous studies, consistent with results from earlier modeling [17]. We note that in our model, a given biomass of herbivorous consumers corresponds to constant per unit-cover grazing rates on dense turf algae and macroalgae (*g*_*T*_*θ* and *g*_*M*_*θ*, respectively). Thus, if the proportional cover of either algal group increases, then the corresponding grazing rate increases, representing a greater encounter rate between consumers and algal cover. This is different to the analytical model of Mumby et al. (2007) [32], in which the per unit-cover grazing rate on macroalgae decreases with the total proportional cover of algae, reflecting lower efficiency in grazing because of the greater areal coverage of algae. Despite these differences, both models are capable of exhibiting alternative stable states and associated hysteresis, because in both models increased algal cover has detrimental effects on coral cover and vice versa, which contribute to feedbacks that can maintain a coral-dominated and an algal-dominated stable state at a given level of herbivorous consumer biomass [32, 33]. Therefore, the qualitative dynamical behavior of the models seems robust to the way grazing is represented, although future work would be required to assess the quantitative differences in dynamical behavior and consequences for the strength of bottom-up versus top-down effects on coral and algal proportional covers.

**Fig 4:**
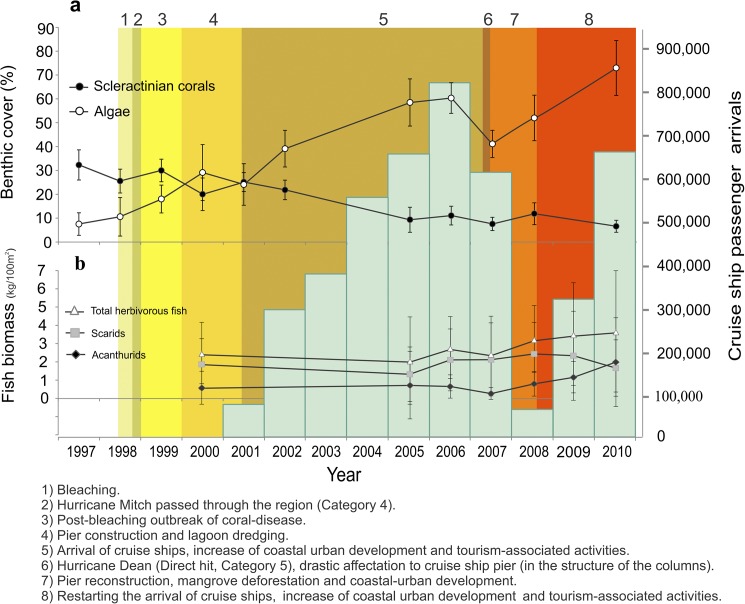
Phase shift on Mahahual reef system, Yucatan coast, Mexico (data from spur and groove system on reef slope, 12 m; and terrace 18 m). a) The phase shift, with increasing algal cover and declining coral cover (means and standard deviations), realized over 14 years. Coral cover did not decline by much in the face of major but discrete regional stress events and disturbances such as elevated temperatures (phase 1); Hurricane Mitch (2); post-bleaching outbreak of coral-disease (3); and pier construction and lagoon dredging (4). Rather, coral decline was associated with increased coastal-urban development, tourism-associated activities, and pier reconstruction and associated dredging (phases 5, 7 and 8), combined with a fierce hurricane (6). b) Biomass densities (means and standard deviations) of the two main groups of herbivorous fish (scarids and acanthurids) and the total biomass density of these two groups were relatively constant over the same period, while tourism increased dramatically, as indicated by cruise ship passenger arrivals. There was a drop in the number of passengers after 2006 due to the deterioration of Costa Maya’s pier after Hurricane Dean, which required two years to rebuild.

This view is supported by our observations of a shift from coral to algal dominance on reefs at Mahahual off the Yucatan coast in eastern Mexico (Fig 4A), in which there was an increase of algal cover but little change in herbivorous fish biomass (Fig 4B). These findings are consistent with previous meso-scale observations at the Mesoamerican Reef [28]. During 1998–2000, bleaching, hurricanes, disease and port works did not cause major changes in benthic cover on the Mahahual reef system. However, from 2000–2003, coral cover decreased and algal cover increased in concert with a massive increase in tourist visitations and the construction of a cruise ship pier. Satellite imagery has revealed that the coastal landscape of Mahahual lost 85 ha of vegetation cover from 2000 to 2006, induced in large part by the construction of the cruise ship pier, hotels and restaurants, while the reefscape suffered a loss of 43 ha of live coral cover [21]. In 2000, the reef lagoon was dredged to extend the area of the surrounding village and allow the construction of hotels near the beach to receive cruise passengers; by 2001, 25 hotels with a combined bed capacity of 50 to 100 rooms were already built in the village. The effects of this sudden coastal development on coral reefs may have been exacerbated by bleaching events and hurricanes [21]. However, while the mean biomass of herbivorous fishes (2 kg/100m^2^) falls within the range of biomass values observed on moderately fished sites along the Mesoamerican barrier reef [61,30], the biomass of herbivores did not show significant variation during the period of the phase shift where coral and algal covers underwent drastic changes (2000–2005; Fig 4, S2 and S3 Tables), indicating that grazing pressure (per unit-cover grazing rates as conceptualized in our model and discussed above) has remained relatively constant. In the past (1955 to 1979) [62] and until recently, local fishermen have largely targeted predatory fish on the reefs at Mahahual and the surrounding areas, with minimal fishing of herbivorous fishes [62–65].

Despite the lack of time-series on nutrient concentrations in Mahahual, there is correlational evidence from other studies that tourism visitations led to increased nitrogen inputs into the coastal marine environments of northern Quintana Roo, which is the most developed part of the Mexican Caribbean coast [26,66,67]. While Mahahual, located in the southern part of Quintana Roo, arguably exhibits lower rates of sewage-derived nitrogen inputs when compared to areas of northern Quintana Roo with a greater intensity of tourism development [23,26], recent point data [68] have indicated high absolute levels of eutrophication on Mahahual reefs, with very high standing concentrations of ammonium (7.69 μM), nitrates (2.43 μM), and orthophosphates (6.68 μM) [68]. In addition, cruise ship traffic has considerably increased from 37 arrivals in 2000 to 223 in 2010, bringing the number of short-term visitors to an average of 800,000 per year. The amount of sewage pollution potentially generated by tourists undertaking short-term (i.e., daily) excursions to the shore is currently unknown, but could be considerable and is likely to be much higher than in 2000. It is also worth noting that high nutrient concentrations, sewage water and high algal covers have been associated with the incidence of coral diseases [69–74], which at Mahahual doubled in prevalence over a decade concomitant with the increase in tourist activity, with surveys showing that coral diseases affected 12.3% of coral colonies in 2001 (before the pier was in operation) but 25.4% of colonies in 2011 (JR Garza-Pérez, unpublished data).

We acknowledge that because of the lack of time-series for nutrient and sediment levels on Mahahual reefs, we cannot be certain that increased nutrients and sediments were the main drivers of the observed phase shift. The shift could also have been driven by coral mortality caused by dredging the reef lagoon, with algae overgrowing the vacant space; this could happen in the absence of increased nutrients or fishing. Another possibility is that nutrients have increased algal productivity, resulting in macroalgae progressively overgrowing corals, which could happen without dredging and associated sedimentation, and without increased fishing. While it is feasible that the phase shift is the result of multiple processes, it is unlikely that fishing is the proximal cause given the constancy of (statistically non-significant changes in) herbivorous fish biomass (Fig 4B), size and abundance over the 2000–2005 period of the phase shift, where coral cover approximately halved and algal cover more than doubled (S2 and S3 Tables). The same trends were found for the biomass, size and abundance of scarids over the same time period, and also the biomass and size of acanthurids. There was a statistically significant increase in the abundance of acanthurids from 2000 to 2005, but this failed to translate into a significance increase in the total abundance of herbivorous fish (acanthurids and scarids). These results suggest that coastal development is associated with the shift to algal dominance, pointing to the importance of bottom-up processes. Similarly, a study in Quintana Roo coast [28] and a recent study of 85 sites along the Mesoamerican Reef [75] did not find a significant correlation between increasing macroalgal cover and variations in herbivorous fish abundance. In the Mesoamerican Reef study macroalgal cover did not exceed 30–35%, whereas on Quintana Roo reefs macroalgae shifted to 20–80% cover and on Mahahual reef to 40–70% cover, and in both the latter cases with a nearly constant biomass of herbivorous fish during the phase shift. Our empirical data show a progressive shift from coral- to algal-dominance, reflecting a loss of resilience that may have been initiated before our monitoring commenced, as has occurred in other parts of the Mexican Caribbean [28]. Importantly, the shift was not caused by an acute and severe coral mortality event (there was no sudden, sharp decline in coral), and herbivore biomass was approximately constant over the period of coral decline.

The effects of tourism and urban development on coral reefs along Quintana Roo’s coast are increasingly recognized [20,22,25–28,30]. The recent development of further tourism infrastructure of Costa Maya in the southern part of Quintana Roo coast constitutes rapid and uncontrolled coastal development, which clearly represents a threat to persistence of coral cover along the Mexican Caribbean coast. Unregulated land use and poor sewage treatment lead to chronic eutrophication and turbid waters that are detrimental for corals [18], and these negative effects can amplify the impacts of diseases and thermal stress associated with climate change [12,76,77]. This is the situation in Mahahual, Cancún and Riviera Maya coral reefs, which are unprotected areas with weak regulation and poor enforcement of existing (and minimal) standards for sewage and other pollutants.

Our empirical observations and model results suggest that rapid, uncontrolled coastal development can precipitate a shift from a healthy reef system to a degraded state characterized by low coral cover and dominance of algae. A management strategy is required that focuses on both effective watershed management as well as maintenance of functional levels of grazing, to maximize resilience to and the likelihood of recovery from phase shifts to degraded states. Otherwise, the functioning of these important ecosystems will be compromised, as is evident in Mesoamerica currently. More holistic management frameworks are required urgently that explicitly acknowledge and address the effects of both bottom-up and top-down stressors on coral reef systems and their functional groups of organisms. Within this framework, the use of long-term research programs and dynamic process-based models are important for capturing dynamic non-linearities and feedbacks [17,32,33,78,79], and for identifying critical levels of stressors that would likely result in coral-algal phase shifts, and to enable management strategy evaluation.

## Supporting information

S1 Table Model parameters and the locations pertaining to data used for parameterization, with corresponding references. To increase readability, the row colors for the parameters alternate between white and gray.
(DOC)

S2 Table Results of PERMANOVA analysis and Pairwise tests of temporal trends in Length (cm), Abundance (number of individuals), and Biomass (kg/100 m^2^) for different categories of herbivorous fish at Mahahual reefs, over the period corresponding to the observed coral-algal phase shift (2000–2010). **The categories are Total herbivorous fish (scarids and scanthurids), scarids and scanthurids.** The pairwise comparisons between the years 2000 and 2005 (referred to in the main text) are highlighted in gray. Df = degrees of freedom; SS = sum of squares; MS = mean sum of squares; Pseudo-F = F value by permutation. Bold face indicates statistical significance (P < 0.05); P-values are based on 10,000 Monte-Carlo samplings (P (MC)).
(DOCX)

S3 Table Average and Standard Deviation (SD) of Length (cm), Abundance (number of individuals) and Biomass (kg/100 m^2^) of Total herbivorous fish (acanthurids and scarids combined), acanthurids and scarids at Mahahual reefs, for years during the observed coral-algal phase shift (DOCX)
