# Supplementary material for: A coral-algal phase shift in Mesoamerica not driven by changes in herbivorous fish abundance
Source: PLoS One. 2017 Apr 26;12(4):e0174855. doi: 10.1371/journal.pone.0174855 (PMC5405933; doi:10.1371/journal.pone.0174855)
Supplement: S1 Table — To increase readability, the row colors for the parameters alternate between white and gray. (DOC) [file pone.0174855.s001.doc]

**S1 Table. Model parameters and the locations pertaining to data used for parameterization, with corresponding references.** To increase readability, the row colors for the parameters alternate between white and gray.

| **Model parameter** | **Location(s) pertaining to data used for parameterization** | **Reference(s)** |
| --- | --- | --- |
| $d_{C}$: coral mortality rate | St. Croix, U.S. Virgin Islands | [1] |
|  | Florida, U.S. | [2] |
| $l_{C}^{b}$, $l_{C}^{s}$: coral endogenous and exogenous recruitment rates over space | Curaçao; Florida, U.S.; Panama (Caribbean coast) | [3] |
|  | 263 sites across the Caribbean | [4] |
|  | Rio Bueno, Jamaica | [5] |
|  | Florida, U.S. | [2] |
|  | Barbados; Bermuda; Jamaica; St. Croix, U.S. Virgin Islands; other unspecified locations in Caribbean | [6] |
|  | Belize | [7] |
| $r_{C}$: coral growth rate over space | Jamaica | [8] |
|  | Curaçao; Florida, U.S.; Panama (Caribbean coast) | [3] |
|  | Belize; Guatemala (Caribbean coast); Honduras (Caribbean coast); Mexico (Caribbean coast) | [9] |
|  | Curaçao; Florida, U.S.; Jamaica; Virgin Islands | [10] |
| $\alpha_{C}$: coral growth rate over turf algae relative to space | No data available  (full theoretical range of 0–1 explored) | |
| $\varepsilon_{C}$: coral recruitment rate over turf algae relative to space | Outdoor aquarium at Orpheus Island Research Station, Australia | [11] |
| $\zeta_{T}$: turf algae growth rate over space | Unspecified | [12] |
|  | Caribbean (unspecified) | [7], [13] |
| $g_{T}$,$g_{M}$: maximum grazing rates on turf algae and macroalgae | Pioneer Bay, Australia | [14], [15] |
|  |  | |
|  | New Caledonia | [16] |
|  | Belize | [13] |
|  | Diet data from 91 coral reef herbivorous fish species, 57 of which were from the western Atlantic (Jamaica; St. John, U.S. Virgin Islands) | [17] |
| $\theta$: proportion of maximum grazing rates on turf algae and macroalgae that is realized | St. John, U.S. Virgin Islands | [18] |
|  | Puerto Rico | [19] |
|  | 302 sites across the western Atlantic | [20] |
|  | New Caledonia | [16] |
|  | Navassa Island (Carribean) | [21] |
|  | Belize | [13] |
|  | Bahamas | [22] |
|  | Belize | [23] |
|  |  |  |
| $r_{M}$: macroalgae growth rate over space | Belize | [24] |
| $\beta_{M}$: negative effect of macroalgae on coral growth rates | Honduras (Caribbean coast) | [25] |
|  | Florida, U.S. | [26] |
| $\gamma_{MC}$, $\gamma_{MT}$: macroalgae growth rates over corals and turf algae relative to space | Belize; Guatemala (Caribbean coast); Honduras (Caribbean coast); Mexico (Caribbean coast) | [9] |
|  | Florida, U.S. | [26] |
|  | Curaçao | [27] |

**Full references**

1. Bythell JC, Gladfelter EH, Bythell M (1993) Chronic and catastrophic natural mortality

of three common Caribbean reef corals. Coral Reefs 12: 143–152.

2. Lirman D (2003) A simulation model of the population dynamics of the branching coral

*Acropora palmata* - effects of storm intensity and frequency. Ecological Modelling 161:

169–182.

3. Edmunds PJ (2007) Evidence for a decadal-scale decline in the growth rates of juvenile

scleractinian corals. Marine Ecology Progress Series 341: 1–13.

4. Gardner TA, Côté IM, Gill JA, Grant A, Watkinson AR (2003) Long-term region-wide

declines in Caribbean corals. Science 301: 958–960.

5. Hughes TP (1984) Population dynamics based on individual size rather than age: a general

model with a reef coral example. The American Naturalist 123: 778–795.

6. Langmead O, Sheppard C (2004) Coral reef community dynamics and disturbance: a

simulation model. Ecological Modelling 175: 271–290.

7. Mumby PJ (2006) The impact of exploiting grazers (Scaridae) on the dynamics of Caribbean

coral reefs. Ecological Applications 16: 747–769.

8. Chornesky EA, Peters EC (1987) Sexual reproduction and colony growth in the

scleractinian coral *Porites astreoides*. Biological Bulletin 172: 161–177.

9. García-Salgado M, Camarena-Luhrs T, Gold G, Vasquez M, Galland G, Nava G, Alarcó G,

Ceja V (2006) 2004-5 Mesoamerican Barrier Reef Systems Project Synoptic Data. Belize

City.

10. Huston M (1985) Variation in coral growth rates with depth at Discovery Bay, Jamaica.

Coral Reefs 4: 19–25.

11. Birrell CL, McCook LJ, Willis BL (2005) Effects of algal turfs and sediment on coral

settlement. Marine Pollution Bulletin 51: 408–414.

12. McClanahan TR (2002) The near future of coral reefs. Environmental Conservation 29: 460–

483.

13. Mumby PJ, Hedley JD, Zychaluk K, Harborne AR, Blackwell PG (2006) Revisiting the

catastrophic die-off of the urchin *Diadema antillarum* on Caribbean coral reefs: fresh

insights on resilience from a simulation model. Ecological Modelling 196: 131–148.

14. Bellwood DR, Hughes TP, Hoey AS (2006) Sleeping functional group drives coral-reef

recovery. Current Biology 16: 2434–2439.

15. Hughes TP, Rodrigues MJ, Bellwood DR, Ceccarelli D, Hoegh-Guldberg O, McCook L,

Moltschaniwskyj N, Pratchett MS, Steneck RS, Willis B (2007) Phase shifts, herbivory,

and the resilience of coral reefs to climate change. Current Biology 17: 360–365.

16. Letourneur Y, Kulbicki M, Labrosse P (1998) Spatial structure of commercial reef fish

communities along a terrestrial runoff gradient in the northern lagoon of New Caledonia.

Environmental Biology of Fishes 51: 141–159.

17. Russ GR, St. John J (1988) Diets, growth rates and secondary production of herbivorous

coral reef fishes. Proceedings of the Sixth International Coral Reef Symposium 2:37–43.

18. Edmunds PJ (2002) Long-term dynamics of coral reefs in St. John, US Virgin Islands. Coral

Reefs 21: 357–367.

19. Irizarry-Soto E (2006) Abundance, composition and survivorship of juvenile corals in the

southwestern Puerto Rico, La Parguera. Masters. University of Puerto Rico, Mayaguez.

20. Kramer PA (2003) Synthesis of coral reef health indicators for the western Atlantic: results

of the AGRRA program (1997–2000). Atoll Research Bulletin 496: 1–58.

21. Miller MW, Gerstner CL (2002) Reefs of an uninhabited Caribbean island: fishes, benthic

habitat, and opportunities to discern reef fishery impact. Biological Conservation 106: 37–

44.

22. Ostrander GK, Armstrong KM, Knobbe ET, Gerace D, Scully EP (2000) Rapid transition in

the structure of a coral reef community: The effects of coral bleaching and physical

disturbance. Proceedings of the National Academy of Sciences of the United States of

America 97: 5297–5302.

23. Williams ID, Polunin NVC, Hendrick VJ (2001) Limits to grazing by herbivorous fishes and

the impact of low coral cover on macroalgal abundance on a coral reef in Belize. Marine

Ecology Progress Series 222: 187–196.

24. Mumby PJ, Foster NL, Fahy EAG (2005) Patch dynamics of coral reef macroalgae under

chronic and acute disturbance. Coral Reefs 24: 681–692.

25. Box SJ, Mumby PJ (2007) Effects of macroalgal competition on growth and survival of

juvenile Caribbean corals. Marine Ecology Progress Series 342: 139–149.

26. Lirman D (2001) Competition between macroalgae and corals: effects of herbivore exclusion

and increased algal biomass on coral survivorship and growth. Coral Reefs 19: 392–399.

27. Nugues MM, Bak RPM (2006) Differential competitive abilities between Caribbean coral

species and a brown alga: a year of experiments and a long-term perspective. Marine

Ecology Progress Series 315: 75–86.
