# Supplementary material for: A coral-algal phase shift in Mesoamerica not driven by changes in herbivorous fish abundance
Source: PLoS One. 2017 Apr 26;12(4):e0174855. doi: 10.1371/journal.pone.0174855 (PMC5405933; doi:10.1371/journal.pone.0174855)
Supplement: S2 Table — The categories are Total herbivorous fish (scarids and scanthurids), scarids and scanthurids. The pairwise comparisons between the years 2000 and 2005 (referred to in the main text) are highlighted in gray. Df = degrees of freedom; SS = sum of squares; MS = mean sum of squares; Pseudo-F = F value by permutation. Bold face indicates statistical significance (P < 0.05); P-values are based on 10,000 Monte-Carlo samplings (P (MC)). (DOCX) [file pone.0174855.s002.docx]

**S2 Table. Results of PERMANOVA analysis and Pairwise tests of temporal trends in Length (cm), Abundance (number of individuals), and Biomass (kg/100 m^2^) for different categories of herbivorous fish at Mahahual reefs, over the period corresponding to the observed coral-algal phase shift (2000-2010). The categories are Total herbivorous fish (scarids and scanthurids), scarids and scanthurids.** The pairwise comparisons between the years 2000 and 2005 (referred to in the main text) are highlighted in gray. Df = degrees of freedom; SS = sum of squares; MS = mean sum of squares; Pseudo-F = F value by permutation. Bold face indicates statistical significance (P < 0.05); P-values are based on 10,000 Monte-Carlo samplings (P (MC)).

| **Category** | **Source** | **Test:Term** | **Effect** | **t** | **Df** | **SS** | **MS** | **F** | **Perms** | **P (MC)** |
| --- | --- | --- | --- | --- | --- | --- | --- | --- | --- | --- |
| **Total herbivorous** | **Length** |  | **Year** |  | **6** | **1339.4** | **223.24** | **4.9087** | **9935** | **0.0001** |
|  |  |  | **Residuals** |  | **161** | **7321.9** | **45.478** |  |  |  |
|  |  |  | **Total** |  | **167** | **8661.3** |  |  |  |  |
|  |  | PAIR-WISE: Year | A2000, A2005 | 1.6475 |  |  |  |  | 9903 | 0.1087 |
|  |  |  | A2000, A2006 | 1.1068 |  |  |  |  | 9915 | 0.2743 |
|  |  |  | A2000, A2007 | 0.6444 |  |  |  |  | 9922 | 0.5977 |
|  |  |  | A2000, A2008 | 2.8794 |  |  |  |  | 9917 | **0.0053** |
|  |  |  | A2000, A2009 | 0.7446 |  |  |  |  | 9904 | 0.4759 |
|  |  |  | A2000, A2010 | 3.8851 |  |  |  |  | 9929 | **0.0004** |
|  |  |  | A2005, A2006 | 3.1818 |  |  |  |  | 9903 | **0.0025** |
|  |  |  | A2005, A2007 | 0.9466 |  |  |  |  | 9915 | 0.3698 |
|  |  |  | A2005, A2008 | 5.9582 |  |  |  |  | 9925 | **0.0001** |
|  |  |  | A2005, A2009 | 2.7799 |  |  |  |  | 9885 | **0.0068** |
|  |  |  | A2005, A2010 | 6.2000 |  |  |  |  | 9912 | **0.0001** |
|  |  |  | A2006, A2007 | 1.0412 |  |  |  |  | 9902 | 0.3051 |
|  |  |  | A2006, A2008 | 1.9383 |  |  |  |  | 9915 | 0.0534 |
|  |  |  | A2006, A2009 | 0.4357 |  |  |  |  | 9899 | 0.6691 |
|  |  |  | A2006, A2010 | 3.2239 |  |  |  |  | 9916 | **0.0021** |
|  |  |  | A2007, A2008 | 1.7346 |  |  |  |  | 9914 | 0.0798 |
|  |  |  | A2007, A2009 | 0.9088 |  |  |  |  | 9914 | 0.3736 |
|  |  |  | A2007, A2010 | 2.4081 |  |  |  |  | 9928 | **0.0134** |
|  |  |  | A2008, A2009 | 2.5151 |  |  |  |  | 9913 | **0.0143** |
|  |  |  | A2008, A2010 | 2.1477 |  |  |  |  | 9917 | **0.0315** |
|  |  |  | A2009, A2010 | 3.6602 |  |  |  |  | 9920 | **0.0006** |
|  | **Abundance** |  | **Year** |  | **6** | **8568.5** | **1428.1** | **7.0329** | **9925** | **0.0001** |
|  |  |  | **Residuals** |  | **161** | **32692** | **203.06** |  |  |  |
|  |  |  | **Total** |  | **167** | **41261** |  |  |  |  |
|  |  | PAIR-WISE: Year | A2000, A2005 | 1.5571 |  |  |  |  | 9939 | 0.1142 |
|  |  |  | A2000, A2006 | 1.3867 |  |  |  |  | 9947 | 0.1627 |
|  |  |  | A2000, A2007 | 0.2770 |  |  |  |  | 9928 | 0.8615 |
|  |  |  | A2000, A2008 | 4.4512 |  |  |  |  | 9940 | **0.0002** |
|  |  |  | A2000, A2009 | 1.1941 |  |  |  |  | 9949 | 0.2298 |
|  |  |  | A2000, A2010 | 1.2137 |  |  |  |  | 9939 | 0.2233 |
|  |  |  | A2005, A2006 | 0.8346 |  |  |  |  | 9949 | 0.4266 |
|  |  |  | A2005, A2007 | 1.7450 |  |  |  |  | 9940 | 0.0746 |
|  |  |  | A2005, A2008 | 2.7095 |  |  |  |  | 9931 | **0.0061** |
|  |  |  | A2005, A2009 | 0.6332 |  |  |  |  | 9935 | 0.5947 |
|  |  |  | A2005, A2010 | 2.8936 |  |  |  |  | 9951 | **0.0031** |
|  |  |  | A2006, A2007 | 1.4950 |  |  |  |  | 9930 | 0.1329 |
|  |  |  | A2006, A2008 | 4.4356 |  |  |  |  | 9923 | **0.0001** |
|  |  |  | A2006, A2009 | 0.2863 |  |  |  |  | 9938 | 0.8643 |
|  |  |  | A2006, A2010 | 2.9318 |  |  |  |  | 9949 | **0.0043** |
|  |  |  | A2007, A2008 | 5.1279 |  |  |  |  | 9933 | **0.0001** |
|  |  |  | A2007, A2009 | 1.3093 |  |  |  |  | 9917 | 0.1926 |
|  |  |  | A2007, A2010 | 1.3412 |  |  |  |  | 9944 | 0.1716 |
|  |  |  | A2008, A2009 | 3.7786 |  |  |  |  | 9933 | **0.0001** |
|  |  |  | A2008, A2010 | 6.3436 |  |  |  |  | 9949 | **0.0001** |
|  |  |  | A2009, A2010 | 2.6113 |  |  |  |  | 9947 | **0.0072** |
|  | **Biomass** |  | **Year** |  | **6** | **15356** | **2559.4** | **6.5128** | **9929** | **0.0001** |
|  |  |  | **Residuals** |  | **161** | **63268** | **392.97** |  |  |  |
|  |  |  | **Total** |  | **167** | **78624** |  |  |  |  |
|  |  | PAIR-WISE: Year | A2000, A2005 | 0.6410 |  |  |  |  | 9950 | 0.5872 |
|  |  |  | A2000, A2006 | 2.9851 |  |  |  |  | 9937 | **0.0028** |
|  |  |  | A2000, A2007 | 1.2549 |  |  |  |  | 9932 | 0.2017 |
|  |  |  | A2000, A2008 | 7.3122 |  |  |  |  | 9938 | **0.0001** |
|  |  |  | A2000, A2009 | 1.6152 |  |  |  |  | 9951 | 0.0909 |
|  |  |  | A2000, A2010 | 1.7921 |  |  |  |  | 9932 | 0.0721 |
|  |  |  | A2005, A2006 | 2.0939 |  |  |  |  | 9948 | **0.0319** |
|  |  |  | A2005, A2007 | 0.7991 |  |  |  |  | 9933 | 0.5357 |
|  |  |  | A2005, A2008 | 5.6987 |  |  |  |  | 9946 | **0.0001** |
|  |  |  | A2005, A2009 | 0.9442 |  |  |  |  | 9945 | 0.3713 |
|  |  |  | A2005, A2010 | 1.0817 |  |  |  |  | 9940 | 0.2896 |
|  |  |  | A2006, A2007 | 1.4308 |  |  |  |  | 9919 | 0.1320 |
|  |  |  | A2006, A2008 | 4.4727 |  |  |  |  | 9938 | **0.0002** |
|  |  |  | A2006, A2009 | 1.0630 |  |  |  |  | 9946 | 0.2984 |
|  |  |  | A2006, A2010 | 1.1035 |  |  |  |  | 9923 | 0.2750 |
|  |  |  | A2007, A2008 | 3.7216 |  |  |  |  | 9938 | **0.0001** |
|  |  |  | A2007, A2009 | 0.7046 |  |  |  |  | 9936 | 0.6221 |
|  |  |  | A2007, A2010 | 0.9516 |  |  |  |  | 9941 | 0.4047 |
|  |  |  | A2008, A2009 | 4.5666 |  |  |  |  | 9943 | **0.0001** |
|  |  |  | A2008, A2010 | 5.2729 |  |  |  |  | 9941 | **0.0001** |
|  |  |  | A2009, A2010 | 0.3659 |  |  |  |  | 9939 | 0.8625 |
| **Scarids** | **Length** |  | **Year** |  | **6** | **2226.5** | **371.08** | **2.8382** | **9933** | **0.0051** |
|  |  |  | **Residuals** |  | **161** | **21050** | **130.75** |  |  |  |
|  |  |  | **Total** |  | **167** | **23276** |  |  |  |  |
|  |  | PAIR-WISE: Year | A2000, A2005 | 0.7253 |  |  |  |  | 9925 | 0.5217 |
|  |  |  | A2000, A2006 | 1.9153 |  |  |  |  | 9927 | **0.0461** |
|  |  |  | A2000, A2007 | 1.1872 |  |  |  |  | 9905 | 0.237 |
|  |  |  | A2000, A2008 | 3.3302 |  |  |  |  | 9944 | **0.0008** |
|  |  |  | A2000, A2009 | 1.8873 |  |  |  |  | 9918 | 0.0565 |
|  |  |  | A2000, A2010 | 1.6939 |  |  |  |  | 9922 | 0.087 |
|  |  |  | A2005, A2006 | 2.6649 |  |  |  |  | 9898 | **0.0108** |
|  |  |  | A2005, A2007 | 1.4371 |  |  |  |  | 9926 | 0.143 |
|  |  |  | A2005, A2008 | 6.2020 |  |  |  |  | 9906 | **0.0001** |
|  |  |  | A2005, A2009 | 2.6554 |  |  |  |  | 9924 | **0.0106** |
|  |  |  | A2005, A2010 | 2.0445 |  |  |  |  | 9905 | **0.034** |
|  |  |  | A2006, A2007 | 0.6540 |  |  |  |  | 9918 | 0.5849 |
|  |  |  | A2006, A2008 | 2.4558 |  |  |  |  | 9919 | **0.0177** |
|  |  |  | A2006, A2009 | 0.1293 |  |  |  |  | 9923 | 0.9368 |
|  |  |  | A2006, A2010 | 1.1574 |  |  |  |  | 9914 | 0.241 |
|  |  |  | A2007, A2008 | 1.7689 |  |  |  |  | 9895 | 0.0693 |
|  |  |  | A2007, A2009 | 0.6616 |  |  |  |  | 9907 | 0.5801 |
|  |  |  | A2007, A2010 | 0.8242 |  |  |  |  | 9879 | 0.4322 |
|  |  |  | A2008, A2009 | 2.7635 |  |  |  |  | 9913 | **0.0088** |
|  |  |  | A2008, A2010 | 1.1811 |  |  |  |  | 9823 | 0.2451 |
|  |  |  | A2009, A2010 | 1.2135 |  |  |  |  | 9898 | 0.2174 |
|  | **Abundance** |  | **Year** |  | **6** | **12234** | **2039** | **8.4558** | **9931** | **0.0001** |
|  |  |  | **Residuals** |  | **161** | **38823** | **241.14** |  |  |  |
|  |  |  | **Total** |  | **167** | **51057** |  |  |  |  |
|  |  | PAIR-WISE: Year | A2000, A2005 | 0.8703 |  |  |  |  | 9935 | 0.4041 |
|  |  |  | A2000, A2006 | 0.5847 |  |  |  |  | 9935 | 0.6361 |
|  |  |  | A2000, A2007 | 0.5880 |  |  |  |  | 9941 | 0.6197 |
|  |  |  | A2000, A2008 | 2.1855 |  |  |  |  | 9944 | **0.0216** |
|  |  |  | A2000, A2009 | 1.0812 |  |  |  |  | 9945 | 0.2835 |
|  |  |  | A2000, A2010 | 3.6029 |  |  |  |  | 9935 | **0.001** |
|  |  |  | A2005, A2006 | 0.9426 |  |  |  |  | 9929 | 0.3501 |
|  |  |  | A2005, A2007 | 1.4461 |  |  |  |  | 9928 | 0.1483 |
|  |  |  | A2005, A2008 | 1.8711 |  |  |  |  | 9939 | 0.0657 |
|  |  |  | A2005, A2009 | 2.0443 |  |  |  |  | 9923 | **0.0409** |
|  |  |  | A2005, A2010 | 5.2567 |  |  |  |  | 9940 | **0.0001** |
|  |  |  | A2006, A2007 | 0.7063 |  |  |  |  | 9930 | 0.4981 |
|  |  |  | A2006, A2008 | 2.8612 |  |  |  |  | 9930 | **0.0059** |
|  |  |  | A2006, A2009 | 1.1048 |  |  |  |  | 9929 | 0.2721 |
|  |  |  | A2006, A2010 | 4.4704 |  |  |  |  | 9940 | **0.0001** |
|  |  |  | A2007, A2008 | 3.0557 |  |  |  |  | 9924 | **0.0025** |
|  |  |  | A2007, A2009 | 0.5066 |  |  |  |  | 9933 | 0.6533 |
|  |  |  | A2007, A2010 | 3.3637 |  |  |  |  | 9934 | **0.0004** |
|  |  |  | A2008, A2009 | 4.0141 |  |  |  |  | 9923 | **0.0001** |
|  |  |  | A2008, A2010 | 7.0248 |  |  |  |  | 9933 | **0.0001** |
|  |  |  | A2009, A2010 | 3.5371 |  |  |  |  | 9931 | **0.0008** |
|  | **Biomass** |  | **Year** |  | **6** | **18461** | **3076.9** | **4.5592** | **9912** | **0.0001** |
|  |  |  | **Residuals** |  | **161** | **108650** | **674.87** |  |  |  |
|  |  |  | **Total** |  | **167** | **127120** |  |  |  |  |
|  |  | PAIR-WISE: Year | A2000, A2005 | 1.0013 |  |  |  |  | 9941 | 0.3665 |
|  |  |  | A2000, A2006 | 1.7779 |  |  |  |  | 9950 | **0.0425** |
|  |  |  | A2000, A2007 | 1.0571 |  |  |  |  | 9932 | 0.3336 |
|  |  |  | A2000, A2008 | 4.5004 |  |  |  |  | 9959 | **0.0001** |
|  |  |  | A2000, A2009 | 0.8147 |  |  |  |  | 9949 | 0.5211 |
|  |  |  | A2000, A2010 | 0.1614 |  |  |  |  | 9949 | 0.9927 |
|  |  |  | A2005, A2006 | 1.8954 |  |  |  |  | 9957 | 0.0505 |
|  |  |  | A2005, A2007 | 1.3758 |  |  |  |  | 9942 | 0.1489 |
|  |  |  | A2005, A2008 | 6.2734 |  |  |  |  | 9946 | **0.0001** |
|  |  |  | A2005, A2009 | 0.7289 |  |  |  |  | 9958 | 0.5644 |
|  |  |  | A2005, A2010 | 1.0944 |  |  |  |  | 9947 | 0.2992 |
|  |  |  | A2006, A2007 | 0.8086 |  |  |  |  | 9932 | 0.5144 |
|  |  |  | A2006, A2008 | 3.4063 |  |  |  |  | 9955 | **0.0005** |
|  |  |  | A2006, A2009 | 1.2046 |  |  |  |  | 9938 | 0.2278 |
|  |  |  | A2006, A2010 | 1.7284 |  |  |  |  | 9951 | 0.0523 |
|  |  |  | A2007, A2008 | 3.1059 |  |  |  |  | 9941 | **0.0008** |
|  |  |  | A2007, A2009 | 0.7471 |  |  |  |  | 9937 | 0.5748 |
|  |  |  | A2007, A2010 | 0.9994 |  |  |  |  | 9938 | 0.3741 |
|  |  |  | A2008, A2009 | 4.4674 |  |  |  |  | 9947 | **0.0001** |
|  |  |  | A2008, A2010 | 4.2674 |  |  |  |  | 9951 | **0.0001** |
|  |  |  | A2009, A2010 | 0.8547 |  |  |  |  | 9951 | 0.4951 |
| **Acanthurids** | **Length** |  | **Year** |  | **6** | **1872.8** | **312.13** | **1.6644** | **9941** | **0.1159** |
|  |  |  | **Residuals** |  | **161** | **30193** | **187.53** |  |  |  |
|  |  |  | **Total** |  | **167** | **32065** |  |  |  |  |
|  | **Abundance** |  | **Year** |  | **6** | **15140** | **2523.3** | **10.062** | **9934** | **0.0001** |
|  |  |  | **Residuals** |  | **161** | **40373** | **250.77** |  |  |  |
|  |  |  | **Total** |  | **167** | **55513** |  |  |  |  |
|  |  | PAIR-WISE: Year | A2000, A2005 | 3.2746 |  |  |  |  | 9858 | **0.0008** |
|  |  |  | A2000, A2006 | 3.7319 |  |  |  |  | 9797 | **0.0003** |
|  |  |  | A2000, A2007 | 1.6615 |  |  |  |  | 8427 | 0.0962 |
|  |  |  | A2000, A2008 | 7.1795 |  |  |  |  | 9943 | **0.0001** |
|  |  |  | A2000, A2009 | 4.6969 |  |  |  |  | 9887 | **0.0001** |
|  |  |  | A2000, A2010 | 3.3242 |  |  |  |  | 9708 | **0.0005** |
|  |  |  | A2005, A2006 | 0.6587 |  |  |  |  | 9909 | 0.5848 |
|  |  |  | A2005, A2007 | 2.1574 |  |  |  |  | 9822 | **0.0227** |
|  |  |  | A2005, A2008 | 2.9704 |  |  |  |  | 9948 | **0.0021** |
|  |  |  | A2005, A2009 | 0.9713 |  |  |  |  | 9938 | 0.3518 |
|  |  |  | A2005, A2010 | 0.3098 |  |  |  |  | 9943 | 0.9034 |
|  |  |  | A2006, A2007 | 2.2800 |  |  |  |  | 9788 | **0.0211** |
|  |  |  | A2006, A2008 | 4.2686 |  |  |  |  | 9934 | **0.0002** |
|  |  |  | A2006, A2009 | 1.6131 |  |  |  |  | 9912 | 0.103 |
|  |  |  | A2006, A2010 | 0.3800 |  |  |  |  | 9915 | 0.8107 |
|  |  |  | A2007, A2008 | 6.2491 |  |  |  |  | 9936 | **0.0001** |
|  |  |  | A2007, A2009 | 3.5387 |  |  |  |  | 9909 | **0.0007** |
|  |  |  | A2007, A2010 | 2.0732 |  |  |  |  | 9801 | **0.032** |
|  |  |  | A2008, A2009 | 2.2703 |  |  |  |  | 9934 | **0.0224** |
|  |  |  | A2008, A2010 | 3.5096 |  |  |  |  | 9927 | **0.0002** |
|  |  |  | A2009, A2010 | 1.2582 |  |  |  |  | 9908 | 0.2078 |
|  | **Biomass** |  | **Year** |  | **6** | **24065** | **4010.8** | **5.0309** | **9921** | **0.0001** |
|  |  |  | **Residuals** |  | **161** | **128350** | **797.23** |  |  |  |
|  |  |  | **Total** |  | **167** | **152420** |  |  |  |  |
|  |  | PAIR-WISE: Year | A2000, A2005 | 0.7858 |  |  |  |  | 9953 | 0.5492 |
|  |  |  | A2000, A2006 | 1.4678 |  |  |  |  | 9947 | 0.1169 |
|  |  |  | A2000, A2007 | 0.9858 |  |  |  |  | 9943 | 0.3671 |
|  |  |  | A2000, A2008 | 3.3239 |  |  |  |  | 9940 | **0.0002** |
|  |  |  | A2000, A2009 | 2.1946 |  |  |  |  | 9952 | **0.0136** |
|  |  |  | A2000, A2010 | 1.9108 |  |  |  |  | 9947 | **0.0253** |
|  |  |  | A2005, A2006 | 0.7498 |  |  |  |  | 9946 | 0.5811 |
|  |  |  | A2005, A2007 | 1.5156 |  |  |  |  | 9948 | 0.0896 |
|  |  |  | A2005, A2008 | 3.2491 |  |  |  |  | 9929 | **0.0003** |
|  |  |  | A2005, A2009 | 1.9097 |  |  |  |  | 9959 | **0.0256** |
|  |  |  | A2005, A2010 | 1.7126 |  |  |  |  | 9956 | **0.0493** |
|  |  |  | A2006, A2007 | 2.3427 |  |  |  |  | 9948 | **0.0046** |
|  |  |  | A2006, A2008 | 3.2596 |  |  |  |  | 9943 | **0.0013** |
|  |  |  | A2006, A2009 | 1.6540 |  |  |  |  | 9928 | 0.0669 |
|  |  |  | A2006, A2010 | 1.6430 |  |  |  |  | 9942 | 0.0716 |
|  |  |  | A2007, A2008 | 4.8190 |  |  |  |  | 9958 | **0.0001** |
|  |  |  | A2007, A2009 | 3.4428 |  |  |  |  | 9944 | **0.0001** |
|  |  |  | A2007, A2010 | 2.9896 |  |  |  |  | 9953 | **0.0002** |
|  |  |  | A2008, A2009 | 1.5963 |  |  |  |  | 9922 | 0.0854 |
|  |  |  | A2008, A2010 | 1.2755 |  |  |  |  | 9937 | 0.1858 |
|  |  |  | A2009, A2010 | 0.8760 |  |  |  |  | 9955 | 0.4643 |
|  |  |  |  |  |  |  |  |  |  |  |
