# Supplementary material for: A coral-algal phase shift in Mesoamerica not driven by changes in herbivorous fish abundance
Source: PLoS One. 2017 Apr 26;12(4):e0174855. doi: 10.1371/journal.pone.0174855 (PMC5405933; doi:10.1371/journal.pone.0174855)
Supplement: S3 Table — (DOCX) [file pone.0174855.s003.docx]

**S3 Table. Average and Standard Deviation (SD) of Length (cm), Abundance (number of individuals) and Biomass (kg/100 m^2^) of Total herbivorous fish (acanthurids and scarids combined), acanthurids and scarids at Mahahual reefs, for years during the observed coral-algal phase shift**

| **Total herbivorous combined** | | | | | | |
| --- | --- | --- | --- | --- | --- | --- |
| **Year** | **Length Avg** | **SD** | **Abundance Avg** | **SD** | **Biomass Avg** | **SD** |
| 2000 | 16.097 | 3.590 | 20.200 | 14.461 | 2.500 | 1.677 |
| 2005 | 13.747 | 3.349 | 19.227 | 14.538 | 2.091 | 2.387 |
| 2006 | 16.603 | 4.052 | 14.889 | 4.797 | 2.803 | 1.689 |
| 2007 | 16.376 | 4.759 | 11.912 | 6.132 | 2.444 | 2.073 |
| 2008 | 18.375 | 3.349 | 15.367 | 6.904 | 3.272 | 1.821 |
| 2009 | 16.665 | 2.911 | 20.250 | 10.027 | 3.539 | 2.803 |
| 2010 | 21.088 | 5.188 | 13.042 | 9.309 | 3.687 | 3.311 |
|  |  |  |  |  |  |  |
| **Acanthurids** | | | | | | |
| **Year** | **Length Avg** | **SD** | **Abundance Avg** | **SD** | **Biomass Avg** | **SD** |
| 2000 | 14.108 | 7.343 | 3.103 | 2.623 | 0.607 | 0.902 |
| 2005 | 12.345 | 4.391 | 8.341 | 13.088 | 0.752 | 1.842 |
| 2006 | 14.947 | 3.700 | 5.583 | 3.931 | 0.674 | 0.635 |
| 2007 | 11.488 | 5.054 | 3.618 | 2.559 | 0.289 | 0.319 |
| 2008 | 15.078 | 3.680 | 6.717 | 5.378 | 0.819 | 0.668 |
| 2009 | 15.679 | 4.125 | 9.667 | 7.528 | 1.185 | 0.856 |
| 2010 | 17.868 | 5.931 | 7.750 | 7.859 | 2.006 | 2.423 |
|  |  |  |  |  |  |  |
| **Scarids** | | | | | | |
| **Year** | **Length Avg** | **SD** | **Abundance Avg** | **SD** | **Biomass Avg** | **SD** |
| 2000 | 14.787 | 4.839 | 17.200 | 12.848 | 1.893 | 1.395 |
| 2005 | 14.729 | 4.576 | 10.886 | 6.700 | 1.339 | 1.494 |
| 2006 | 17.503 | 5.999 | 9.306 | 4.432 | 2.129 | 1.690 |
| 2007 | 17.880 | 6.246 | 8.294 | 5.441 | 2.155 | 1.999 |
| 2008 | 20.609 | 4.654 | 8.650 | 4.839 | 2.453 | 1.723 |
| 2009 | 17.739 | 4.516 | 10.583 | 6.107 | 2.354 | 2.430 |
| 2010 | 21.255 | 8.756 | 5.292 | 4.544 | 1.681 | 1.540 |
